# Myocardial injury in infants ventilated on the paediatric intensive care unit: a case control study

**DOI:** 10.1186/cc5040

**Published:** 2006-09-11

**Authors:** Simon J Clark, Michael Eisenhut, Dorothea Sidaras, Stephen W Hancock, Paul Newland, Kent Thorburn

**Affiliations:** 1 Royal Liverpool Children's NHS Trust, Eaton Road, Liverpool L12 2AP, UK

## Abstract

**Introduction:**

Cardiac troponin T (cTnT) has been used to assess prevalence of myocardial injury in critically ill children. The majority of studies investigated patients undergoing cardiac surgery. Myocardial injury has been associated with increased mortality. Our objectives were to investigate whether cTnT levels are elevated in infants without congenital heart disease admitted to the paediatric intensive care unit (PICU) and whether levels are associated with increased disease severity.

**Methods:**

We measured cTnT in consecutive infants (<12 months old) without congenital heart disease admitted to the PICU and healthy infants. The Paediatric Index of Mortality (PIM) score was determined in patients on the PICU.

**Results:**

We recruited 107 infants: 47 infants admitted to the PICU and 60 healthy controls. Controls were, with a median (interquartile range (IQR)) age of 20 (12 to 34) weeks, significantly older than cases, with a median age of 6.5 (0.3 to 20.6) weeks. CTnT levels were, with a median (IQR) of 18 (10 to 60) pg/ml, significantly higher in admissions to the PICU than in controls, with a median level of 10 (10 to 10) pg/ml (95th centile of 20 pg/ml) (*p* < 0.001). There was a significant positive correlation (r = 0.41, *p* = 0.004) between PIM score and cTnT levels. Admissions under one month old had higher cTnT levels than older patients (*p* = 0.013) but the PIM score was not significantly different between them. When corrected for age and weight the correlation of PIM and cTnT was no longer significant.

**Conclusion:**

Infants on the PICU in the neonatal period have higher cTnT levels compared to older infants despite not having more severe disease.

## Introduction

Troponin is an inhibitory protein complex forming part of the contractile apparatus of all striated muscle, including the heart. Specific forms of the troponin subunits T, C and I exist in different muscle types. Cardiac specific troponins T and I have become established as the gold standard biochemical markers for myocardial necrosis [1,2]. The third generation assays for cardiac troponin T (cTnT) are now so sensitive and specific that a concept of minimal myocardial damage has arisen [1]. These marginal elevations of cTnT are associated with increased hospital morbidity and mortality in adult patients following admission [3,4].

There are now increasing numbers of reports of troponin being measured in children as a marker for myocardial injury and disease severity. In healthy older children and neonates (in cord blood) cardiac troponins are undetectable [5,6]. Elevated cardiac troponin levels have been detected in children critically ill with congenital heart disease before and after cardiac surgery [5,7]. In patients without congenital heart disease raised cardiac troponin levels have been found in paediatric intensive care admissions with severe respiratory syncytial virus infection [8] and with meningococcal and other forms of septicemia [9-12]. In meningococcal septicemia cardiac troponin levels have been associated with increased disease severity and cardiac dysfunction [9].

We tested the hypotheses that in critically ill infants without congenital heart disease there is evidence of myocardial injury and that cTnT levels are associated with disease severity.

## Materials and methods

### Patients

We measured cTnT in two groups of infants (less than one year old). The first group comprised critically ill infants admitted to a tertiary paediatric intensive care unit (PICU) at the Royal Liverpool Children's Hospital for ventilatory support. From this group we collected two samples, one on admission and another 24 hours later. All blood sampling was performed at the time of routine phlebotomy or arterial line sampling. We excluded patients admitted for routine cardiac surgery or with a primary cardiac diagnosis.

The second group was a control group of healthy infants having elective invasive procedures, who had one sample taken at the time of routine outpatient phlebotomy, or during day case, or short stay uncomplicated surgery when venous accessed was established. Children with known cardiac abnormalities were excluded from this group.

The local research ethics committee approved the study protocol and valid written parental consent was obtained.

### Data collection

Demographic data were recorded, including the age of the patient on admission, diagnosis and pre-existing illnesses, the Paediatric Index of Mortality (PIM) score [13], ventilation settings, blood gases, requirements for fluid boluses, inotrope infusions, and outcome.

### Sample collection and analysis

Samples were centrifuged, separated and frozen at -20°C until batch analysis was performed. We performed biochemical analysis with an Elecsys 1010 System analyser using the Elecsys Troponin T STAT Immunoassay (Roche Diagnostics GmbH, Mannheim, Germany). This electrochemiluminescent sandwich enzyme linked immunosorbant assay has a lower limit of detection of 10 pg/ml, with minimal cross reactivity with cardiac troponin I of 0.002%, and skeletal troponin T of 0.001% [14]. The coefficient of variation for paired samples was less than 10% across the assay range. The coefficient of variation for the internal quality control was less than 6.4% at two concentrations (100 pg/ml and 5,400 pg/ml).

### Data analysis

cTnT is not normally distributed. Therefore, non-parametric tests were used for this parameter and other continuous data as appropriate; these included Mann-Whitney *U* test, Wilcoxon signed rank test and Spearman's rank correlation coefficient. Chi-square and Fisher's exact test were used for comparison of categorical data as appropriate. Correction for age and weight was performed using partial correlation coefficients and by subgroup analysis. We performed subgroup analysis to compare those admissions under one month old with those over one month old. A *p* value of less than 0.05 was taken as an indicator of a statistically significant difference. Analysis was performed using Arcus BioStat (Biostat Inc., Englewood, NJ, USA) and SPSS (SPSS Inc., Chicago, IL, USA) release 13.0. Undetectable levels of cTnT were assigned the value of the lower limit of the assay, 10 pg/ml.

## Results

### Healthy Infants

Sixty healthy infants were recruited into the study. The cTnT levels had a median (interquartile range) of 10 (10 to 10) pg/ml with a 95th percentile of 20 pg/ml (maximum: 100 pg/ml). Their median age was 20 (12 to 34) weeks, with 48 (77%) of the samples being collected under general anaesthetic. cTnT was detected in six (10%) of the healthy infants. Diagnoses in these six patients were (one patient each): inguinal hernia; skin tag; haemangioma; hypothyroidism; phenylketonuria; and haemolytic anaemia. There was no statistically significant difference between the values from surgical patients or from medical patients within this group. Table 1 shows the list of diagnoses in healthy infants.

### Paediatric intensive care admissions

We included 47 PICU patients who were all intubated and ventilated in our study. We obtained 47 admission samples, which had cTnT levels with a median of 18 (10 to 60) pg/ml. This was statistically significantly higher that the levels of the healthy infants (*p* < 0.001). There was a statistically significant correlation between the PIM score and cTnT (r = 0.41, *p* = 0.004). This relationship was no longer significant once age and weight were corrected for (r = 0.025, *p* = 0.95). The duration of mechanical ventilation was not different between patients with and without elevated cTnT levels, with a median of 3.0 days in both groups (*p* = 0.66).

**Figure 1:**
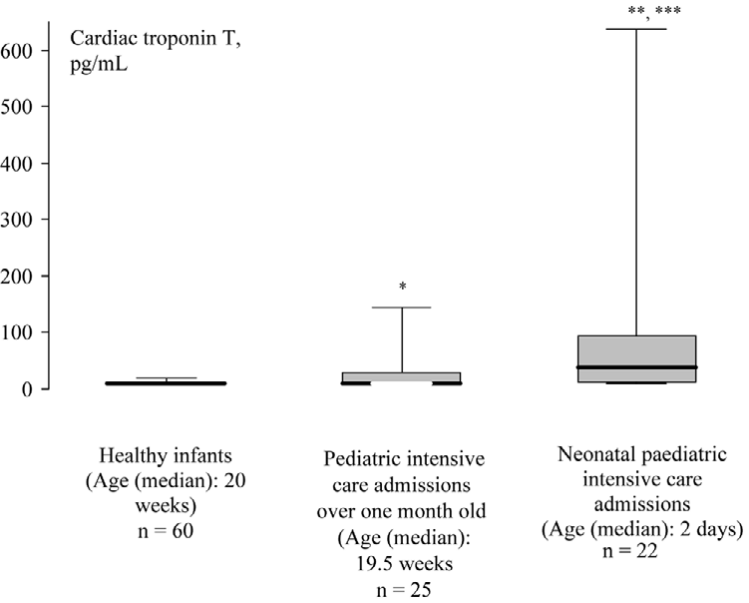
Box and whisker plots for cardiac troponin T values in neonatal and older intensive care admissions along with healthy infants. The boxes are the interquartile ranges, the whiskers are the 5th to 95th percentiles, with the heavy black line representing the median. *p = 0.006 compared with healthy infants; **p = 0.013 compared with older intensive care admissions; ***p < 0.001 compared with healthy infants.

Of the 47 admissions to paediatric intensive care, 22 were infants less than one month old (neonates) and they had statistically significantly higher cTnT levels when compared with the older infants admitted for paediatric intensive care (median 38 (19 to 95) pg/ml versus 10 (10 to 30) pg/ml, *p* = 0.013; Figure 1). There was no correlation between the PIM score and the cTnT levels in the neonates (r = 0.27, *p* = 0.22). In the 25 older paediatric intensive care admissions, the correlation between the PIM score and cTnT remained significant (r = 0.44, *p* = 0.028). However, there was no statistically significant difference between the PIM scores for neonates and older infants on the PICU. One infant in each group required inotropes.

In the neonates on the PICU cTnT levels for only 5/22 were undetectable compared with 17/25 undetectable results in the 25 older infants (*p* = 0.003 using Fisher's exact test). The older paediatric intensive care admissions' levels of cTnT were statistically significantly higher than the control population (*p* = 0.006; Figure 1).

Table 2 gives demographic details for these two subgroups. There was an excess of surgical patients among the neonates (17/22 versus 6/25, *p* < 0.001 with Fisher's exact test). Table 3 gives the list of diagnoses for both groups. We included a total of seven patients with sepsis, one neonate and six older infants. Elevated cardiac troponin levels were found in two of these seven infants. There were two deaths; one from meningitis and one from secondary pneumonia following respiratory syncytial virus infection and both patients had undetectable cTnT on admission.

### Paired samples

As the samples were measured secondary to clinical analysis there was resulting attrition arising from small volume collection and/or second samples not being collected and only 35 paired samples were obtained. There were no changes in the 16 paired samples from admissions under one month old (median 47 (11 to 84) pg/ml on admission and 43 (10 to 92) pg/ml 24 hours later). There were also no changes in the levels from the 19 paired samples in older infants (median 10 (10 to 28) pg/ml on admission and 10 (10 to 10) pg/ml 24 hours later). The neonatal levels were statistically significantly higher on admission and 24 hours later (*p* = 0.049 and *p* = 0.007, respectively) compared with the older infants admissions' levels.

## Discussion

Admissions to paediatric intensive care had significantly raised cTnT levels compared to controls and this elevation persisted for at least 24 hours. There was a significant correlation between the cTnT levels and the disease severity score, but this did not persist when age and weight were corrected for. Neonates admitted to paediatric intensive care had significantly higher cTnT levels compared with the older intensive care admissions, but despite this there was no statistically significant difference in the critical illness score between these two subgroups.

### Neonates on PICU

In neonates elevation of cTnT may relate to perinatal stress factors [5]. Minor elevations of around 25 pg/ml are seen more commonly between 48 and 96 hours after delivery [15]. This has been associated with exposure to tocolytic agents before delivery [16] and perinatal hypoxia [17]. In neonates with moderate asphyxia elevated cTnT levels of up to 110 pg/ml on day 7 decreasing to about 30 pg/ml on day 15 of life have been observed [18]. These elevations are not associated with adverse outcomes, nor do they seem to be affected by any demographic variables [6,15]. Therefore, it is possible that newborns admitted to a PICU (not admitted for neonatal care) would need a different reference range from older admissions [16].

**Figure 2:**
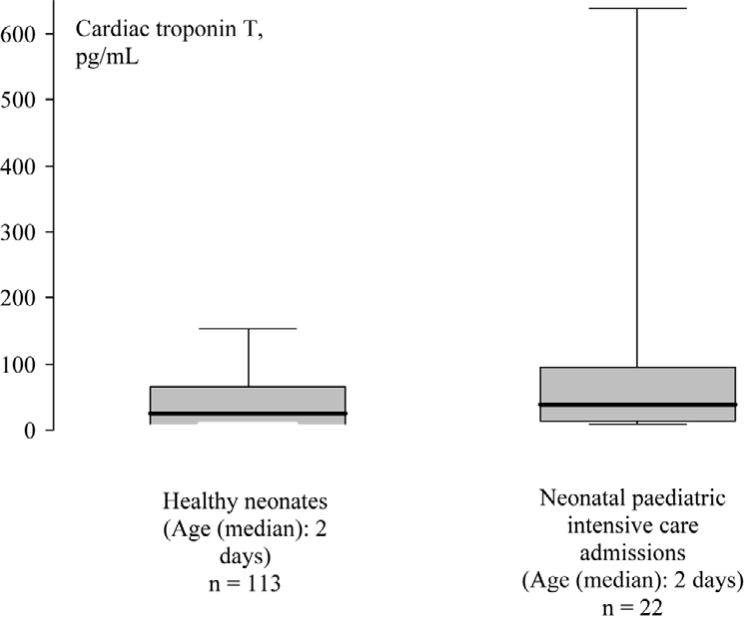
Box and whisker plots for cardiac troponin T values in healthy neonates (data taken from [9]) and neonatal paediatric intensive care admissions. The boxes are the interquartile ranges, the whiskers are the 5th to 95th percentiles, with the heavy black line representing the median.

In the healthy infants, our control group, the youngest patient was nearly one month old. Many of the youngest admissions to paediatric intensive care in this study were within the first week after delivery, attested by their median (interquartile range) age of 2 (1 to 5) days. It may be that the very youngest paediatric intensive care admissions are better compared with healthy neonatal controls. In our previous study looking at 113 healthy neonates sampled at a median of 2 (3 to 4) days, we found cTnT levels of a median of 25 (10 to 62) pg/ml [15]. These healthy neonatal levels are not statistically significantly different from the levels of the 22 neonates admitted for paediatric intensive care, with a median of 38 (19 to 95) pg/ml (Figure 2). Most of the neonatal admissions to paediatric intensive care were not suffering from conditions commonly associated with hypoxia (for example, gastoschisis, tracheoesophageal fistula, exomphalos). So it may be that the levels of troponin on admission and 24 hours later reflect the transient neonatal rise of troponin following delivery. However, 5 babies were admitted with potentially profound causes of hypoxia: 4 babies with a congenital diaphragmatic hernia with troponin levels of 10, 58, 79, and 236 pg/ml and a baby with persistent pulmonary hypertension of the newborn and a troponin level of 720 pg/ml. It would not be unreasonable to assume that the highest levels in those infants indicated myocardial hypoxia secondary to their presenting problem rather than just perinatal stress.

### Infants over one month

Admissions over one month old had minimal, but statistically significant, elevations of cTnT compared with healthy infants. This very minor elevation compared to adults with critical illness [18] probably reflects the cardiac reserve seen in children compared with adults during critical illness, due to pristine coronary arteries and the different clinical pathways that lead to paediatric intensive versus adult admissions. However, 4 infants had admission troponin levels above 100 pg/ml: 2 cases of septicaemia (105 and 120 pg/ml); and 2 cases of subglottic stenosis requiring airway management (150 and 160 pg/ml). Elevated cTnT levels have been reported in children with septicemia before [9]. It is possible that these four children with high cTnT levels would have had poor end organ oxygenation leading to diffuse myocardial injury. This was supported by the persistence of the correlation between the disease severity score and the cardiac troponin T level in infants above one month of age.

### Paired samples

Levels of cTnT did not alter significantly following admission. This may reflect the release kinetics of cTnT, or the type of myocardial injury seen in these babies. In a full thickness myocardial infarct troponin starts to rise within two hours of the onset of chest pain and reaches diagnostic levels by 12 hours later, but it can still be detected up to two weeks later as troponin slowly leaks out of the necrotic area [1]. Many paediatric intensive care admissions receive intensive care prior to their admission (sometimes for many hours), either in peripheral hospitals or neonatal units. It is possible that sampling at admission to paediatric intensive care occurs several hours into their critical illness at a point when troponin levels have already started to rise, which is why little change was seen from admission to 24 hours later.

### Healthy infants

In most of our healthy infants under one year old cTnT was undetectable and this is in keeping with other studies [15].

### Study limitations

Due to consecutive recruitment without age matching, controls were significantly older than infants on the PICU. There was, however, no significant difference between the age of controls and infants beyond the neonatal period on the PICU with their significantly higher cTnT levels. This illustrated that myocardial injury was not only related to age.

We did not have routine echocardiographic assessment performed in our study population and, therefore, we do not have markers of direct myocardial performance. Eisenhut and colleagues [8] measured cTnT levels in babies admitted with severe respiratory syncytial virus and did not find a relationship with echocardiographic markers of performance. Infants with elevated cTnT levels were significantly younger that patients without. The levels of troponin in this study were of similar magnitude (50 (38 to 68) pg/ml). However, echocardiographic assessment was made around 48 hours following admission. This could allow any myocardial dysfunction to resolve following delivery of intensive care. Therefore, the opportune time to perform echocardiographic assessment would be within the first few hours of admission to the PICU.

Another possible concern is the ontogeny of troponins. Bodor and colleagues [19] demonstrated expression of cTnT in foetal skeletal muscle up to 20 weeks of gestation. However, they could not demonstrate cTnT in healthy mature human skeletal muscle by western blot.

## Conclusion

Infants on a PICU in the neonatal period have higher cTnT levels compared to older infants despite not having more severe disease. cTnT is not a suitable marker of disease severity in neonates on a PICU. cTnT in critically ill infants over one month old may better relate to disease severity scores.

## Key messages

• Evidence of myocardial injury with elevated cTnT on admission was seen in 17/22 neonates and 8/25 older infants ventilated on the PICU.

• cTnT levels were higher in neonates compared to older infants ventilated on the PICU despite not having more severe disease.

• cTnT levels correlated significantly with disease severity in infants older than one month. This is compatible with myocardial injury being related to the acute illness leading to PICU admission in these infants.

## Abbreviations

BP = blood pressure; cTnT = cardiac troponin T; PICU = paediatric intensive care unit; PIM = Paediatric Index of Mortality.

## Competing interests

The authors declare that they have no competing interests.

## Authors' contributions

JS Clark participated in the design of the study, performed data analysis and participated in writing the paper. ME participated in recruiting of patients, data collection, data analysis and interpretation and writing of the paper. DS participated in recruiting patients, and collecting and interpreting the data. SWH participated in the design of the study, recruiting patients and collecting data. KT participated in the design of the study, recruiting of patients and collection and analysis of data. PN participated in analysis of laboratory samples and data collection and analysis.

## Figures and Tables

**Table 1:** Diagnoses in 60 healthy infants

	No. of infants (percent)
Male	36 (60)
Age in weeks (median, IQR)	20 (12 to 34)
Orthopaedic procedures	6 (10)
Neurosurgical procedures	2 (3)
Circumcisions	5 (8)
Magnetic resonance scan under anaesthetic	1 (2)
Cleft lip repairs	5 (8)
Inguinal hernia repairs	8 (13)
Other abdominal operations	7 (12)
Plastic/laser surgery	6 (10)
Pyeloplasty	1 (2)
Plastic/laser surgery	6 (10)
Cataracts	1 (2)
Medical patients	12 (20)

IQR, interquartile range.

**Table 2:** Characteristics of paediatric intensive care admissions below and above one month of age

	Neonates <1 month old (*n *= 22)	Older infants >1 month old (*n *= 25)	*p* value
Age (weeks)	0.3 (0.1–0.7)	20 (13–26)	<0.001^a^
Weight (kg)	2.7 (2.2–3.2)	5.8 (4.0–8.0)	<0.001^a^
Male (n (percent))	14 (64)	16 (64)	0.780^b^
Surgical (n (percent))	17 (52)	6 (24)	<0.001^c^
PIM (percent)	7.9 (3.2–10.8)	3.9 (1.5–7.5)	0.052^a^
Volume resuscitation (ml/kg)	10 (0–30)	0 (0–20)	0.450^a^
Systolic BP on admission (mmHg)	71 (62–77)	105 (85–120)	<0.001^a^
Length of ventilation (days)	3 (2–9)	3 (2–5)	0.779^a^

^a^Mann-Whitney test; ^b^Chi square-test; ^c^Fishers exact test. PIM, paediatric index of mortality. Except where specified, all values in parentheses represent interquartile ranges.

**Table 3:** Diagnoses for paediatric intensive care admissions

	Neonatal paediatric intensive care admissions (*n *= 22)	Older paediatric intensive care babies (*n *= 25)
Congenital diaphragmatic hernia	4 (18)	0
Neurosurgical	1 (5)	3 (12)
Major abdominal surgery	7 (31)	2 (8)
Airway obstruction	1 (5)	4 (16)
Tracheoesophageal fistula	4 (17)	0
Sacrococcygeal teratoma	1 (5)	0
Sepsis	1 (5)	6 (24)
Bronchiolitis	0	6 (24)
Bacterial lower respiratory tract infection	2 (9)	3 (12)
Persistent pulmonary hypertension of the newborn	1 (5)	0
Pulmonary haemorrhage	0	1 (4)

Values are numbers, with percentages in parentheses.
